# Unveiling the Role of Sialylation-Related LncRNAs in Lung Adenocarcinoma: A New Prognostic Model

**DOI:** 10.2174/0113892029382696251125102036

**Published:** 2026-04-15

**Authors:** Guixue Yang, Juncheng Yu, Xufeng Deng, Jigang Dai, Quanxing Liu

**Affiliations:** 1 Department of Thoracic Surgery, Xinqiao Hospital, Army Medical University (Third Military Medical University), Chongqing 400037, China;; 2 Hematopoietic Acute Radiation Syndrome Medical and Pharmaceutical Basic Research Innovation Center, Ministry of Education of the People’s Republic of China, Chongqing 400037, China

**Keywords:** Lung adenocarcinoma, sialylation, lncRNA, prognostic signature genes, risk signature model

## Abstract

**Background:**

Lung adenocarcinoma (LUAD) patients have a poor overall survival rate. Sialylation is closely related to lung cancer progression, and long non-coding RNAs (lncRNAs) are crucial in tumorigenesis. However, how sialylation and lncRNAs affect LUAD is unclear.

**Methods:**

This study utilized the TCGA-LUAD dataset, GSE31210, GSE131907, and a set of 109 sialylation-related genes (SRGs). Differential expression analysis was performed on TCGA-LUAD tumor and control samples to identify differentially expressed lncRNAs (DE-lncRNAs). Spearman correlation analysis with SRGs was then conducted to screen for sialylation-related lncRNAs (SRDLs). Risk-signature models were constructed using univariate and multivariate Cox regression analyses, followed by a proportional hazards (PH) test. Independent prognostic factors were identified, and a nomogram was developed to predict 1-, 3-, and 5-year survival. Gene expression in key cell populations was examined using GSE131907, and GSEA, tumor immune microenvironment assessment, and drug sensitivity analyses were performed. Finally, prognostic genes were validated using RT-qPCR.

**Results:**

A total of 139 SRDLs were identified. A four-gene risk model (LINC00115, LINC00173, LINC00968, LINC01352) was constructed. Both the risk score and T stage were independent prognostic factors. Single-cell analysis of GSE131907 identified eight cell types, with myeloid cells emerging as key contributors. The high-risk group was associated with cell-cycle–related pathways, whereas the low-risk group was enriched in neuroactive ligand–receptor interaction pathways. Immune infiltration analysis revealed multiple significant correlations, and tumor mutation burden scores differed between the two groups. RT-qPCR validated the observed gene expression patterns.

**Discussion:**

The four-lncRNA sialylation signature predicted LUAD outcomes and pointed to myeloid cells as therapeutic targets. Prospective validation and mechanistic studies are, however, needed.

**Conclusion:**

Four sialylation-associated lncRNAs were identified in LUAD, enabling the construction of an effective risk model. Myeloid cells were highlighted as key contributors, offering valuable insights for personalized treatment and prognostic intervention.

## 1 INTRODUCTION

Lung adenocarcinoma (LUAD), a predominant subtype of non-small cell lung cancer (NSCLC), presents substantial public health challenges due to its high incidence and poor prognosis [1]. The five-year survival rate for patients with LUAD remains significantly low, primarily due to late-stage diagnoses and the disease's inherent biological heterogeneity, which complicates treatment approaches and drives high healthcare costs [2]. Current treatment options, including chemotherapy, radiotherapy, targeted therapies, and surgical resection, often demonstrate limited efficacy due to tumor resistance mechanisms and inter-patient variability [3]. Moreover, the adverse effects of conventional therapies remain a critical concern. Consequently, there is an urgent need to identify novel prognostic biomarkers to improve early detection and enable the development of tailored treatment strategies for patients with LUAD.

Post-translational modifications (PTMs) play a pivotal role in modulating protein function by covalently adding or removing small molecular groups [4]. More than 200 distinct PTMs have been identified, influencing various cellular processes, such as metabolism, signaling, and protein stability [5, 6]. Among these, sialylation has emerged as a key modification linked to immune evasion, metastasis, tumor growth, and chemoresistance [7]. Sialylation, the final step in glycosylation, involves the addition of sialic acid to the terminal ends of carbohydrate chains on glycoproteins and glycolipids [8]. Abnormal sialylation, commonly observed in cancer, is regulated by two enzyme groups: sialyltransferases, which catalyze sialylation, and sialidases, which facilitate desialylation [9]. Recent findings highlight hypersialylation and enhanced sialyltransferase activity as emerging hallmarks of cancer, contributing to tumor progression, metastasis, immune evasion, and treatment resistance [9]. Furthermore, the accumulation of sialic acid diminishes the effectiveness of chemotherapy and radiation therapy, promoting immune evasion. It also facilitates cancer spread by enhancing cell motility, tissue penetration, and vascular development [10, 11].

Long non-coding RNAs (lncRNAs) are RNA molecules longer than 200 nucleotides that do not encode proteins but are involved in various gene regulatory mechanisms, including chromatin remodeling, transcriptional regulation, and modulation of signaling pathways [12, 13]. Recent studies have highlighted the potential of specific lncRNAs as prognostic markers in LUAD, suggesting that their expression levels may correlate with tumor characteristics and patient survival outcomes [7, 14]. For instance, the research study by Zhang *et al.* [15] indicated that elevated CRNDE expression was strongly associated with advanced tumor dedifferentiation, higher TNM classification, lymphatic spread, reduced radiotherapy effectiveness, and significantly poorer survival rates. Similarly, Yang *et al.* [16] discovered that lncRNA PCAT6 acts as a miR-545-3p sponge, facilitating LUAD progression. Moreover, several studies have shown that lncRNAs regulate sialylation and contribute to cancer pathogenesis. For example, downregulation of lncRNA MEG3 affects EGFR sialylation by upregulating ST3Gal1, which subsequently inhibits the PI3K-AKT signaling pathway in renal cell carcinoma [17]. In another study, overexpression of lncRNA ST3GAL6-AS1 was linked to enhanced invasiveness in multiple myeloma through the upregulation of sialyltransferase ST3GAL6 [18]. Collectively, these findings underscore the critical role of lncRNAs in sialylation and their potential as diagnostic markers and therapeutic targets in cancer [18].

## 2 MATERIALS AND METHODS

### 2.1 Data Source

LUAD-related datasets were sourced from the GEO and GDC databases. The TCGA-LUAD dataset comprised 497 LUAD tumor tissue samples and 58 control lung tissue samples, including patient lncRNA and mRNA data, clinical characteristics, and survival information. The raw lncRNA and mRNA data of LUAD were preprocessed, with the data maintained in raw count format [19]. These LUAD samples were randomly divided into a training set and a test set in a 7:3 ratio (384:113) for subsequent analyses [20-23]. The GSE31210 dataset (platform: GPL570), which included 226 LUAD tumor tissues and 20 control lung tissue samples, served as the validation set. The already standardized expression matrix (log2-transformed gene-level signal values) was downloaded from this dataset. The single-cell dataset, GSE131907 (platform: GPL1691), contained 11 primary LUAD tumor tissues and 11 control lung tissue samples. The single-cell RNA-seq data were normalized by unique molecular identifier (UMI). Additionally, 109 sialylation-related genes (SRGs) were retrieved from MSigDB [24]. A total of 38 immune checkpoints were compiled from previous studies (Table **S1**) [25].

### 2.2 Differential Expression Analysis

Differentially expressed lncRNAs (DE-lncRNAs) were identified through differential expression analysis of tumor and control samples in TCGA-LUAD using DESeq2 (v1.42.0) [26] with |log_2_FC| > 1 and FDR < 0.05. Volcano plots of DE-lncRNAs were generated using ggplot2 (v3.4.4) [27], and heatmaps of the top 50 up- and down-regulated DE-lncRNAs were plotted with ComplexHeatmap (v2.14.0) [28]. To identify sialylation-related differential lncRNAs (SRDLs), Spearman correlation analysis was performed between the 109 SRGs and DE-lncRNAs, selecting genes with stronger correlations as SRDLs.

### 2.3 Construction, Evaluation, and Validation of Risk Signature Models

A novel risk signature model for LUAD was constructed using regression analyses on the training set. First, univariate Cox analysis was conducted on the SRDLs using the survival package (v3.5-7) [29] to identify survival-associated genes (*p* < 0.05). The cox_zph function was then used to perform a proportional hazards (PH) assumption test on these genes (*p* > 0.05). Genes that passed the PH assumption were included in the multifactorial Cox analysis, followed by model adjustment using stepwise regression (STEP() function with the parameter direction set to “both”) to identify prognostic signature genes and construct the risk signature model. The stepwise selection procedure utilized AIC (Akaike information criterion) as the optimization target, wherein variables were iteratively included or excluded to obtain the final model with the lowest AIC value [30].

The risk score for each patient was calculated based on the expression levels of the prognostic signature genes and their corresponding coefficients derived from multivariate Cox regression analysis. The formula was defined as:, where *X_i_* denotes the expression value of gene *i*, and coef*_i_* represents its regression coefficient estimated by the Cox proportional hazards model using maximum partial likelihood. A positive coefficient indicates a risk factor, while a negative coefficient suggests a protective factor. Patients were then stratified into high-risk and low-risk groups based on the optimal cut-off value determined by the survminer R package. This method evaluated the independent contribution of each variable (the expression level of lncRNAs) to the risk event. The absolute value of the coefficient indicated the predictive importance of the variable, while the sign (positive/negative) reflected its positive (risk factor) or negative (protective factor) correlation with the outcome. Patients were divided into risk cohorts based on the optimal risk score threshold. Survival differences (*p* < 0.05) between cohorts were evaluated using Kaplan-Meier (K-M) survival curves and calculating the C-index, plotted with Survminer (v0.4.9) [31]. Additionally, survivalROC (v1.0.3) [32] was used to plot ROC curves at 1, 3, and 5 years to assess the model's predictive accuracy. The model was further validated in both the test set and the validation set.

### 2.4 Hierarchical Analysis

To examine the distribution of risk scores across clinical features, clinical variables, such as gender, age, stage, tumor (T), node (N), and metastasis (M), were considered, and heat maps of prognostic signature genes were generated. Clinical features were then stratified, and differences in risk scores between subtypes were assessed using the Wilcoxon test [33].

### 2.5 Independent Prognostic Analysis and Construction of a Nomogram

To assess whether risk scores could serve as independent predictors of the disease, independent prognostic analyses were performed. Initially, risk scores were incorporated alongside clinical features in univariate Cox regression analyses (*p* < 0.05), followed by PH assumption tests (*p* > 0.05) for factors showing significance. Factors that passed these tests were further examined in multivariate Cox regression analyses to identify independent prognostic factors (*p* < 0.05). Subsequently, nomograms were developed using the rms package (v6.7-1) [31], incorporating significant prognostic variables to estimate the 1-, 3-, and 5-year survival rates of patients. Each factor was assigned a corresponding score, with the total score determining the survival rate, where higher scores indicated better survival outcomes. The model's validity and accuracy were further validated through calibration curves, DCA, and receiver operating characteristic (ROC) curves.

### 2.6 Gene Set Enrichment Analysis (GSEA), Drug Sensitivity Analyses, and Molecular Regulation

To explore the functional and pathway differences between the risk cohorts, GSEA was performed using the clusterProfiler package (v4.7.1.003) [34]. First, differences between risk cohorts were analyzed with DESeq2, calculating log_2_FC and sorting them from largest to smallest. The c2.cp.kegg.v7.5.1.entrez.gmt gene set from the MSigDB repository was employed as the reference gene set for GSEA analysis (*p* < 0.05). Additionally, given the heterogeneity of LUAD, variations in drug sensitivity across patients were assessed. The 138 chemotherapy/targeted therapy drugs were obtained from the Genomics of Drug Sensitivity in Cancer (GDSC, https://www.cancerrxgene.org) database, and the half-maximal inhibitory concentrations (IC_50_) for each patient were calculated using pRRophetic (v0.5) [35]. The Wilcoxon test was applied to evaluate the differences in IC_50_ values between risk groups. Furthermore, miRNAs associated with the prognostic signature genes (lncRNAs) were predicted using the starBase (https://starbase.sysu.edu.cn) database (clipExpNum ≥ 1 and pancancerNum ≥ 3). Next, corresponding mRNAs for the miRNAs were identified in the miRWalk database (http://mirwalk.umm.uni-heidelberg.de), and those supported by TargetScan, miRDB, and experimental verification were selected as key mRNAs. The lncRNA-miRNA-mRNA network was constructed using Cytoscape (v3.10.1) [36].

### 2.7 Analysis of Immune Infiltration and Immunotherapy

CIBERSORT [37] was employed to assess the relative infiltration levels of 22 immune cell types across the risk groups. Disparities in immune cell infiltration between these groups were evaluated using the Wilcoxon test. The relationship between prognostic signature genes and immune cell populations was then explored through Spearman's rank correlation coefficient [38]. To predict the response to immunotherapy, differences in the expression of 38 immune checkpoint inhibitors (ICIs) between risk cohorts were compared using the Wilcoxon test (*p* < 0.05). The correlation between prognostic signature genes and differential ICI expression was also analyzed (|cor|>0.3, *p* < 0.05) [39]. To determine whether the risk profile model could predict the immunotherapeutic potential of LUAD, comparisons of TIDE, TMB, MSI, and KRAS mutation differences between risk cohorts were made (*p* < 0.05).

### 2.8 Single-cell RNA Sequencing (scRNA-seq) Analysis

For single-cell level analysis of prognostic signature gene expression, data from GSE131907 were utilized. The PercentageFeatureSet function of Seurat (v5.0.1) [40] was used to calculate nCount_RNA, nFeature_RNA, and percent.Mt for all samples, ensuring the selection of high-quality cells with gene counts between 200 and 20,000 and mitochondrial gene proportions < 10%. The data were normalized using LogNormalize, and the FindVariableFeatures function identified the top 2,000 most variable genes for subsequent analysis. These genes were subjected to PCA downscaling, with available dimensions determined by the ElbowPlot function. Unsupervised clustering was performed using FindNeighbors and FindClusters functions, and UMAP [41-43] plots (resolution = 0.2) were generated to visualize the results. Different cell types were identified based on marker gene annotation.

### 2.9 Identification of Key Cells and Cellular Communication

To identify key cells, cell type occupancy profiles for tumors and controls were analyzed using the Wilcoxon test. Cells showing higher expression of key mRNAs in differential cells were considered key cells. Additionally, the cellular communication network was investigated using CellChat (v1.6.1) [44].

### 2.10 Annotation of Key Cells and Pseudo-time Analysis

Key cells were further downscaled and clustered for analysis, with the clustering results visualized using t-SNE [45, 46] plots (resolution = 0.3) and annotated by marker genes. Subsequently, pseudo-time analysis was performed on the key cells using Monocle2 (qval < 0.01) (v2.30.1) [47].

### 2.11 Expression Validation of Prognostic Signature Genes

Gene expression data for the prognostic signature genes were obtained from the TCGA-LUAD cohort to assess expression differences between tumor and control samples. Additionally, the expression levels of these genes were validated *via* RT-qPCR. A set of 5 pairs of normal and LUAD tissue samples [48-51] was collected from the Second Affiliated Hospital of Army Medical University. Informed consent was obtained from all subjects, the clinical investigations were conducted in accordance with the principles of the Declaration of Helsinki, and the study was approved by the hospital's ethics committee (approval number 2022-062-01).

Following the manufacturer's protocol, total RNA was extracted from the 10 specimens using TRIzol reagent (Invitrogen, China). RNA concentrations were then measured with the NanoPhotometer N50. cDNA synthesis was performed *via* reverse transcription using the SureScript First-strand cDNA synthesis kit (Servicebio, China). qPCR was conducted on the CFX Connect Thermal Cycler (Bio-Rad, USA). The relative expression levels of lncRNAs were quantified using the 2^-ΔΔCT^ method [52]. Primer sequences are provided in Table **S2**.

### 2.12 Statistical Analysis

All analyses were performed using R software (v4.3.2). Statistical significance was defined as *p* < 0.05, unless otherwise specified.

## 3 RESULTS

### 3.1 Construction of a Risk Signature Model for LUAD Based on Four Prognostic Signature Genes

A total of 1,099 DE-lncRNAs, comprising 751 upregulated and 348 downregulated, were identified between LUAD and control samples in the TCGA-LUAD dataset (Fig. **1A**-**B**). Among them, 139 DE-lncRNAs strongly associated with SRGs were selected as SRDLs based on correlation analysis (Fig. **1C**). Further, 8 survival-associated genes were identified through univariate Cox analysis (*p* < 0.05) (Table **1**), of which 7 passed the PH assumption test, all acting as protective factors (*HR* < 1, *p* < 0.05) (Fig. **1D**). Using multifactorial Cox analysis combined with stepwise regression, four lncRNAs, including LINC00115, LINC00173, LINC00968, and LINC01352, were selected as prognostic signature genes to construct the risk signature model (Table **2**, Fig. **1E**).

### 3.2 Evaluation and Validation of the Effectiveness and Accuracy of the Risk Signature Model

In the training set, the risk score was calculated as follows: RiskScore = LINC00115 × (-0.289) + LINC00173 × (-0.151) + LINC00968 × (-0.166) + LINC01352 × (-0.175). Patients were stratified into different risk cohorts based on the optimal risk score threshold (0.9751). Risk curves showed an increase in patient mortality with higher risk scores (Fig. **2A**). K-M survival curves revealed that patients in the high-risk cohort had significantly lower survival rates, and the C-index was 0.627, indicating that the model had a certain predictive value (Fig. **2B**). Furthermore, the AUC values at 1, 3, and 5 years exceeded 0.6 (Fig. **2C**), indicating good predictive accuracy and validity of the constructed risk signature model. To further validate the prognostic value of the model, it was tested in both the test and validation sets. In both sets, mortality rates increased with higher risk scores (Fig. **2D**, **G**), and K-M survival curves demonstrated substantial survival differences among risk groups (test set, *p* = 0.016, C-index = 0.614; validation set, *p* = 0.0023, C-index = 0.649) (Fig. **2E**, **H**), with AUC values exceeding 0.6 at 1, 3, and 5 years (Fig. **2F**, **I**), confirming the efficacy of the model.

### 3.3 Identification of Independent Prognostic Factors and Nomogram Construction

Heatmap visualization showed that all prognostic signature genes were predominantly expressed in the low-risk group (Fig. **3A**). Stratified analyses revealed that risk scores were higher in patients aged < 60 compared to those ≧ 60, significantly higher in stage II compared to stage I, notably higher in N1 and N2 stages compared to N0, and markedly higher in T3 compared to T1 (Fig. **3B**). Further Cox and PH analyses demonstrated that both risk score and T stage were identified as independent prognostic factors (Fig. **4A**-**C**). Nomograms incorporating risk score and T stage were developed to predict 1-, 3-, and 5-year survival probabilities (Fig. **4D**). The calibration curves for 1-, 3-, and 5-year survival had slopes of 0.7568, 0.3848, and 0.1799, respectively, suggesting the model's optimal prediction at 1 year (Fig. **4E**). Additionally, AUC values for the model at 1, 3, and 5 years exceeded 0.6, further demonstrating its high predictive accuracy (Fig. **4F**). The model also exhibited the highest net benefit compared to T stage and risk score, indicating its diagnostic potential (Fig. **4G**).

### 3.4 Pathways Associated with Risk Cohorts, Molecular Regulation, and Drug Sensitivity

GSEA was performed to explore the functions and pathways associated with high- and low-risk cohorts, revealing distinct patterns. The high-risk cohort was primarily involved in the cell cycle, pyrimidine metabolism, and homologous recombination pathways, while the low-risk cohort was predominantly associated with cytokine-cytokine receptor interactions, the gut immune response for IgA production, and neuroactive ligand-receptor interactions (Fig. **5A**-**B**). Additionally, drug sensitivity was assessed, identifying 39 drugs with significant differences in sensitivity between the risk cohorts (*p* < 0.05). Most of these drugs showed notably reduced sensitivity in the high-risk cohort, including A.443654, PF.4708671, GW843682X, BI.2536, S-Trityl-L-cysteine, and BIBW2992, suggesting that these drugs may have more effective therapeutic outcomes in the low-risk cohort. Only the box plots for the six drugs with the highest significance are presented (Fig. **5C**). Further analysis predicted 11 lncRNAs corresponding to miRNAs from the starBase database, and 51 mRNAs supported by TargetScan, miRDB, and experimental validation were identified from the miRWalk database. A lncRNA-miRNA-mRNA network comprising 62 nodes and 68 edges was constructed (Fig. **5D**).

### 3.5 Prognostic Signature Genes may Influence LUAD Onset and Progression *via* Immune Cells

To explore the role of prognostic signature genes in the immune microenvironment of LUAD, the percentage of 22 immune cell types in different risk cohorts was analyzed (Fig. **6A**). Significant differences in immune cell infiltration were observed between the cohorts, with six immune cells showing notable variations (*p* < 0.05). Naive B cells, resting memory CD4(+) T cells, and resting NK cells had significantly lower infiltration levels in the high-risk cohort, while T follicular helper (Tfh) cells, T regulatory cells (Tregs), and activated NK cells were significantly higher in the high-risk cohort (Fig. **6B**). Correlation analysis revealed a strong positive association between LINC00173 and NK resting cells, a significant negative correlation of LINC00968 with both Tfh cells and Tregs, and a similar negative correlation of LINC01352 with Tfh cells (Fig. **6C**).

### 3.6 Patients in the Low-risk Cohort may have Better Treatment Response

Immunotherapy responses were predicted for patients with LUAD, with 32 ICIs showing significant differences between risk cohorts (*p* < 0.05). Most of these ICIs were higher in the low-risk group, suggesting better immunotherapy responses for patients in this cohort (Fig. **7A**). Moreover, correlation analysis indicated significant negative correlations of LINC00115 with IDO1, IDO2, and LGALS, a positive correlation of LINC00173 with TNFSF14, and a similar positive correlation of LINC01352 with CD28, while LINC00968 showed a negative correlation with both BTNL2 and CD160 (Fig. **7B**). In addition, the TMB score was notably higher in the high-risk cohort (*p* < 0.05) (Fig. **7C**).

### 3.7 Myeloid Cells Considered as Key Cells

In the GSE131907 dataset, after quality control, 79,189 cells were selected for subsequent analysis (Fig. **S1A**). The top 2000 highly variable genes (Fig. **S1B**) and principal components (PCs) were chosen for 30 dimensions (Fig. **S1C**). Clustering of the data yielded 15 cell clusters (Fig. **8A**), which were annotated into 8 distinct cell types: myeloid cells, T lymphocytes, endothelial cells, B lymphocytes, epithelial cells, natural killer (NK) cells, mast cells, and fibroblasts (Fig. **8B**). The bubble plot demonstrated that the marker genes in each cell type were specific (Fig. **8C**). Comparisons between tumor and control groups revealed that T lymphocytes and B lymphocytes were significantly higher in the tumor group, whereas myeloid cells and NK cells were notably lower in the tumor group (Fig. **8D**). Furthermore, 51 key mRNAs were found to be highly expressed in myeloid cells, which were therefore considered key cells for further analysis (Fig. **8E**).

### 3.8 Strong Interactions between Key Cells and Other Cells

Cellular communication analysis revealed interactions among the cell types, with the strongest interactions occurring between myeloid cells and epithelial cells (Fig. **9A**). Furthermore, myeloid cells interacted with seven other cell types, showing particularly strong interactions with epithelial cells, B lymphocytes, and endothelial cells (Fig. **9B**). The ligand-receptor pairs involved in these interactions included MIF-(CD74+CD44), MIF-(CD74+CXCR4), LGALS9-CD44, and LGALS9-CD45 (Fig. **9C**).

### 3.9 Cells Annotated to Seven Different Cell Types

Further downscaling of the key cells identified the top 20 PCs for analysis (Fig. **S2**). Clustering revealed 11 cell clusters, which were annotated into seven distinct cell types: pDC, cDC1, cDC2, mono, macro C1, macro C2, and macro C3 (Fig. **10A**-**B**). Specific marker genes were identified for each cell type (Fig. **10C**). Developmental differentiation trajectories of key cells were also analyzed, revealing seven distinct stages of differentiation from the initial to the final development stages (Fig. **10D**). The distribution of different cell types across these stages of key cell differentiation is presented in Fig. **10D**. Four genes with high expression in the key mRNAs, including MTDH, VCAN, SDHD, and MITF, were selected for further analysis. Among these, VCAN expression increased and then decreased over time across various cell states and subpopulations, whereas MITF expression remained stable without significant changes (Fig. **10E**).

### 3.10 Expression of Prognostic Signature Genes

In the TCGA-LUAD dataset, LINC00115 and LINC00173 were significantly upregulated in tumor samples, while LINC00968 and LINC01352 were notably downregulated (Fig. **11A**). Consistent with this, RT-qPCR results confirmed that LINC00115 and LINC00173 showed significantly higher expression in the tumor group, while LINC00968 displayed significantly lower expression, mirroring the expression patterns observed in TCGA-LUAD. Conversely, LINC01352 did not show a significant difference in expression between the tumor and control groups (Fig. **11B**).

## 4 DISCUSSION

The development of LUAD is influenced by multiple factors, including post-translational protein modifications, oncogene activation, and tumor suppressor gene silencing. Previous research has highlighted the prognostic significance of sialylation-related lncRNAs in cancer [7, 24]. In the present study, four sialylation-associated lncRNAs (LINC00115, LINC00173, LINC00968, and LINC01352) were identified as significantly correlated with LUAD prognosis and notably underexpressed in the high-risk cohort. Through an integrated analysis of single-cell RNA sequencing (scRNA) and transcriptomic data, these lncRNAs emerge as potential prognostic markers and could provide insights into their impact on drug sensitivity and the immune microenvironment. The findings from this study are expected to enhance our understanding of the molecular pathways involved in LUAD progression and contribute to the development of future therapeutic strategies.

The identification of these four prognostic lncRNAs (LINC00115, LINC00173, LINC00968, and LINC01352) significantly deepens our understanding of the biological mechanisms underlying LUAD. It has been shown that LINC00115 is upregulated in LUAD tissues and cells. By modulating Sp3 expression through its interaction with miR-154-3p, LINC00115 knockdown has been found to inhibit LUAD cell growth, invasion, migration, and proliferation [53]. Furthermore, LINC00115 may influence FGF2-mediated oncogenic pathways *via* its interaction with miR-7 [54]. In NSCLC, reduced levels of LINC00173 lead to the accumulation of miR-182-5p, which subsequently impedes cell migration, proliferation, and apoptosis through the AGER/NF-kappaB signaling pathway [55]. Additionally, the LINC00173-SNAIL regulatory axis controls the transcription of the proto-oncogene FHIT in lung carcinoma [56]. LINC00968 suppresses the progression of LUAD *via* the miR-21-5p/SMAD7 pathway [57]. Moreover, prognostic models incorporating lncRNAs, such as LINC01352, have the potential to predict outcomes, tumor mutational load, immune cell infiltration patterns, and responses to immunotherapies and targeted treatments in patients with LUAD [58, 59]. In our study, RT-qPCR confirmed that LINC00115 and LINC00173 exhibited significantly higher expression in tumor samples, whereas LINC00968 showed a significant reduction in expression. These results were consistent with the expression patterns observed in the TCGA-LUAD dataset. Collectively, these lncRNAs represent promising prognostic biomarkers and highlight novel therapeutic possibilities for managing LUAD.

GSEA provided valuable insights into the biological significance of the identified prognostic lncRNAs, shedding light on their role in LUAD. The strong enrichment of cell cycle and pyrimidine metabolism pathways in the high-risk group suggested that these lncRNAs may be linked to LUAD invasiveness. LINC00346, for example, acts as a sponge for miR-30c-2-3p, promoting lung cancer progression by interacting with MYBL2 and modulating the cell cycle regulatory network [60]. The connection with pyrimidine metabolism further underscores a metabolic shift that supports rapid cell proliferation, reinforcing the critical role of metabolic reprogramming in the survival and growth of cancer cells within the tumor microenvironment [61]. Additionally, low-risk lncRNAs were associated with immune-related pathways, such as cytokine-cytokine receptor interactions, suggesting their potential to promote a more favorable immune environment [62]. These findings emphasize the multifaceted nature of LUAD and the need for therapeutic approaches that target both tumor cell proliferation and the immune microenvironment.

The analysis of immune cell infiltration across risk groups revealed significant differences that could influence therapeutic strategies. The reduction in resting NK cells and memory CD4^+^ T cells in the high-risk group indicated a potentially immunosuppressive microenvironment, which may reduce the effectiveness of immunotherapies [63-65]. For patients with LUAD undergoing individualized treatment, a genetic risk model associated with memory CD4^+^ T cells may serve as a reliable predictive indicator [66]. In contrast, the increased presence of regulatory T cells suggested that tumors may employ immune evasion strategies to escape immune surveillance, posing a critical challenge in LUAD management [67, 68]. Understanding the interactions between these immune cell populations and the identified lncRNAs could pave the way for novel combinatorial therapies that enhance immune responses while targeting tumor-promoting pathways [69]. This approach may offer improved outcomes for patients with LUAD, as treatments addressing both tumor biology and the immune landscape could prove more effective than conventional therapies alone.

Immunotherapy has become a cornerstone of LUAD treatment, and the exploration of immune checkpoint interactions has provided essential insights into its application. Lou *et al.* identified six peroxisome-related lncRNAs and demonstrated their significant predictive value in immune function, immune cell infiltration, and TMB levels in LUAD [70]. In our study, the significant correlations between LINC00173 and TNFSF14, as well as LINC01352 and CD28, highlighted the critical role of these lncRNAs in modulating immune checkpoints. Cancer stem-like cells (CSLCs) often develop stronger immune checkpoint responses to evade immune cell death and facilitate tumor growth [71]. The role of FGL1 in regulating immune checkpoint interactions *via* the YY1-FGL1-MYH9 axis is also noteworthy, as FGL1 influences the release of immune-related cytokines and contributes to LUAD cell proliferation and immune modulation [72]. These findings underscore the need for further investigation into the functional roles of these lncRNAs in shaping the immune microenvironment and their potential as therapeutic targets to enhance the effectiveness of immunotherapy in patients with LUAD.

Drug sensitivity analysis provided novel insights into the therapeutic landscape for LUAD [73]. The reduced sensitivity to various chemotherapeutic agents in the high-risk group suggested that these patients may require alternative treatment approaches [63]. A study indicated that A-443654, a potent pan-Akt inhibitor, could be more effective in patients at higher risk for developing LUAD [74]. Additionally, both *in vitro* and *in vivo* studies have demonstrated that the p70S6K-targeting inhibitor PF-4708671 suppresses NSCLC tumor formation [75]. Identifying specific drugs that show higher efficacy in the low-risk cohort may guide clinical decision-making, suggesting that patient stratification based on lncRNA expression profiles could optimize therapeutic regimens [69]. Moreover, understanding the mechanisms by which these lncRNAs influence drug resistance could open pathways for developing combination therapies aimed at reversing resistance and improving treatment outcomes for patients with LUAD. This comprehensive approach cannot only enhance our understanding of LUAD prognostic factors but also support the exploration of personalized therapeutic strategies tailored to individual patient profiles.

In recent years, significant progress has been made in the study of lncRNAs for prognostic prediction in LUAD, with several studies establishing clinically valuable lncRNA signature models through various strategies. Chen *et al.* developed an optimal prognostic model consisting of nine immune-related lncRNAs by applying a selection criterion of the highest average C-index (>0.6) [76]. Fan and colleagues focused on epigenetic regulation and identified three H3K4me3-lncRNA patterns characterized by distinct immune features [77]. Meanwhile, Wang's team revealed the potential role of Tregs and the associated lncRNA PCBP1-AS1 in LUAD diagnosis and treatment [78]. Zhang *et al.* identified six RNA methylation-related lncRNAs to construct a prognostic model through TCGA data, and further integrated TMB, MSI, and drug sensitivity analyses to provide guidance for personalized treatment of LUAD patients [79]. Compared to these findings, our study has highlighted the unique prognostic value of sialylation-related lncRNAs in LUAD. The predictive model we constructed demonstrated stable performance in the training, testing, and validation cohorts (C-index >0.6). Furthermore, using single-cell RNA sequencing, we identified myeloid cells as key effector cells and, through cell-cell communication and pseudotime trajectory analyses, elucidated their dynamic regulatory networks. These findings can provide novel theoretical insights and potential therapeutic targets for understanding LUAD pathogenesis and developing innovative treatment strategies.

However, this study involves several limitations. The lack of experimental validation limits the robustness of the findings, as conclusions drawn from bioinformatics analyses alone may not fully capture the complexity of LUAD biology. Additionally, the relatively small sample size may restrict the generalizability of our results and increase the risk of overfitting in predictive models. Liu *et al.* integrated transcriptomic and proteomic data from multi-ethnic populations to develop a racially adaptable prognostic model for LUAD, revealing that the concordance between mRNA and protein expression across populations was approximately 40-60% [80]. This study underscores the need for future validation of our lncRNA-based prognostic model on large, multi-center cohorts to assess its applicability and robustness. Moreover, potential batch effects inherent in the datasets used may introduce biases that could affect our interpretations. Furthermore, due to current laboratory funding and time constraints, we were unable to conduct functional experiments to investigate the effects of the identified long non-coding RNAs (lncRNAs) on cell proliferation and migration. Future studies could incorporate assays, such as MTT, EdU, wound healing, and transwell migration assays, to validate the functional roles of these lncRNAs and further elucidate their involvement in tumor cell invasion and metastasis.

## CONCLUSION

In conclusion, this study has developed a prognostic risk-scoring model based on four sialylation-associated lncRNAs in LUAD and provided preliminary evidence of their potential involvement in the tumor immune microenvironment. These findings may offer a basis for future exploration of personalized therapeutic strategies and prognostic assessments in LUAD.

## Figures and Tables

**Fig. (1):**
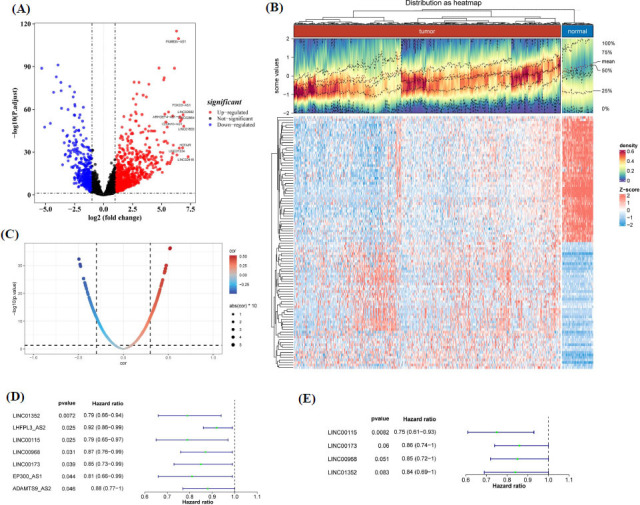
DE-lncRNAs and Cox regression analysis between the normal and tumor groups in the TCGA-LUAD database. (**A**) Volcano plots and (**B**) cluster heatmaps displaying DE-lncRNAs between the normal and tumor groups in LUAD samples. Expression intensity is represented by a color gradient from blue to red, normalized accordingly; (**C**) correlation analysis between DE-lncRNAs and SRGs; (**D**) univariate Cox analysis and (**E**) multivariate Cox analysis of DE-lncRNAs associated with SRGs. The green squares indicate that the hazard ratio (*HR*) value is less than 1, and the line segments on both sides of the square represent the 95% confidence interval (*CI*) of the *HR* value.

**Fig. (2):**
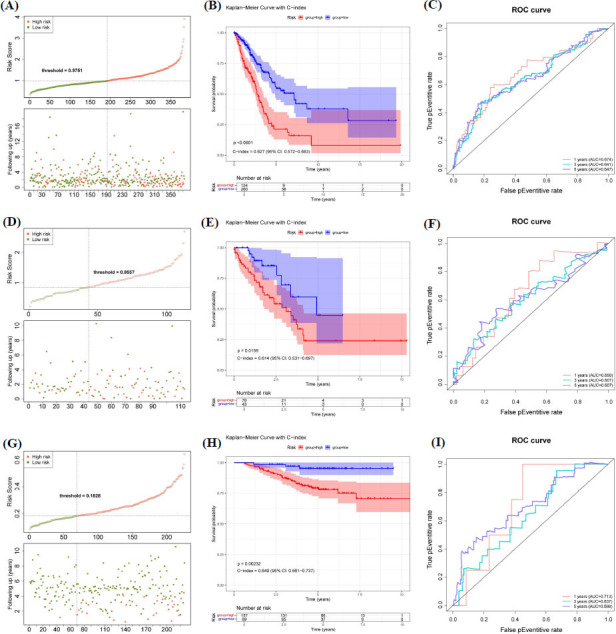
Evaluation and verification of models for risk characterization. (**A-C**) Construction of a risk signature model in the training set, featuring (**A**) risk factor analysis for patients with LUAD (n = 384, cut-off value = 0.9751, with green indicating low-risk and red indicating high-risk groups), (**B**) survival analysis, and (**C**) validation of the risk signature model in the training set; the x-axis denotes specificity (false positive rate), measuring the model’s misclassification rate of negative cases, whereas the y-axis represents sensitivity (true positive rate), quantifying the correct identification rate of positive cases. (**D-I**) Validation of the risk signature model in the test and external sets.

**Fig. (3):**
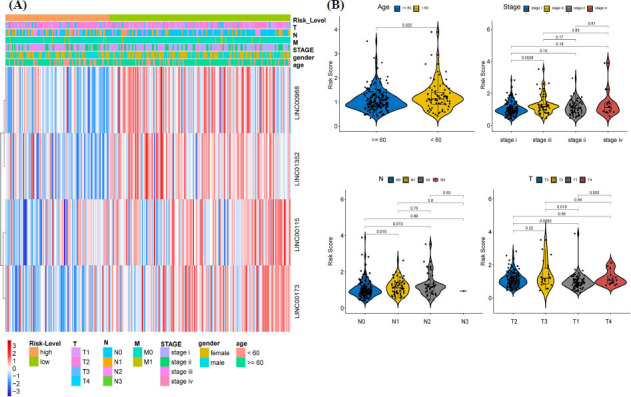
Comparison of clinicopathological characteristics between the high-risk and low-risk groups. (**A**) Heatmaps comparing four differential expression lncRNAs (DE-lncRNAs) and clinicopathological traits between high-risk and low-risk cohorts; (**B**) violin plots illustrating the distribution of risk scores across clinicopathological traits, such as age, N stage, T stage, and TNM stage.

**Fig. (4):**
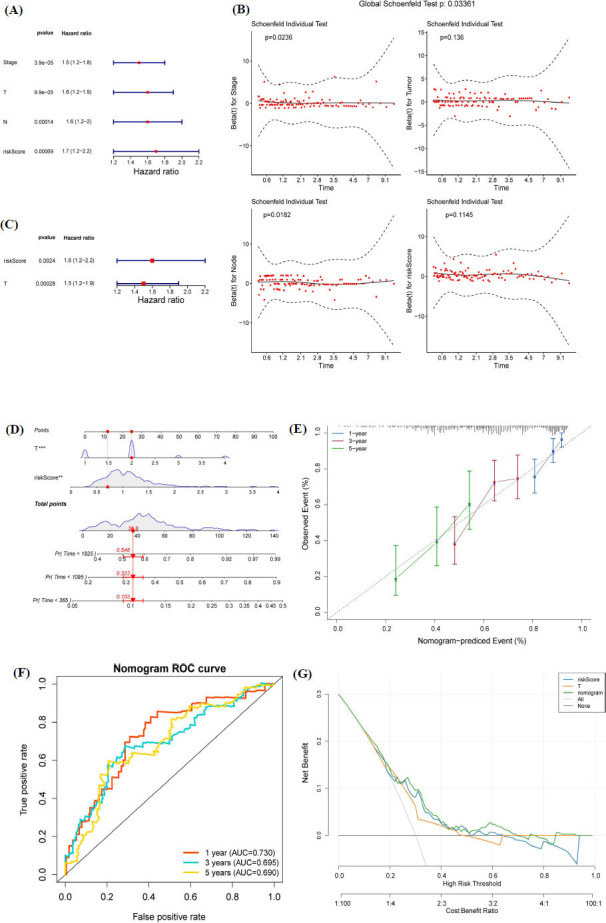
Prognostic value of the risk signature model. (**A**) Univariate Cox analysis; the red squares indicate that the hazard ratio (HR) value is greater than 1, and the line segments on both sides of the square represent the 95% confidence interval of the HR value. (**B**) Proportional hazard (PH) test, and (**C**) multifactorial Cox analysis of risk score and clinicopathological traits. (**D**) Nomogram analysis. The upper section displays the contribution magnitude (regression coefficients) of various clinical factors to the outcome variable. Each factor level is assigned points, with points indicating the individual score for specific clinical factor values. The middle section represents total points, presenting the sum of all individual clinical factor scores. The lower section presents the calculated total points matched against the reference scale to determine the patients’ 1-, 3-, and 5-year survival probabilities. (**E**) Calibration curves. The x-axis represents the predicted probability, indicating the model-calculated risk/event probability, while the y-axis denotes the observed probability, reflecting the true event rate measured experimentally. Closer alignment of the curve to the diagonal indicates better model calibration accuracy. (**F**) Receiver operating characteristic (ROC) curves. (**G**) Decision curve analysis for T stage, risk score, and nomogram. The x-axis represents the threshold probability, while the y-axis shows the net benefit (benefits minus harms). The different curves correspond to distinct clinical prediction models.

**Fig. (5):**
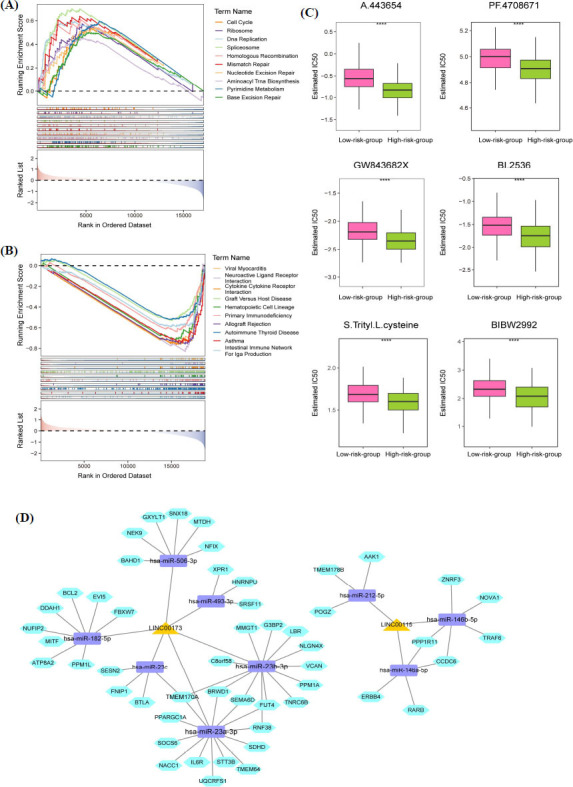
Functional analysis between high- and low-risk groups. (**A, B**) Gene set enrichment analysis (GSEA) between high and low-risk groups; the x-axis represents rank in the ordered dataset, indicating the position of genes after they are sorted according to their degree of association with the phenotype. The y-axis (ordinate) represents enrichment score and ranked list. Enrichment score represents the enrichment degree of the gene set in the ranked gene list, and the ranked list represents the metric value for gene ranking. (**C**) Box plot showing variations in drug sensitivity between high and low-risk groups. (**D**) Regulatory network diagram depicting the relationship between lncRNA and miRNA-mRNA. ****, *p* < 0.0001.

**Fig. (6):**
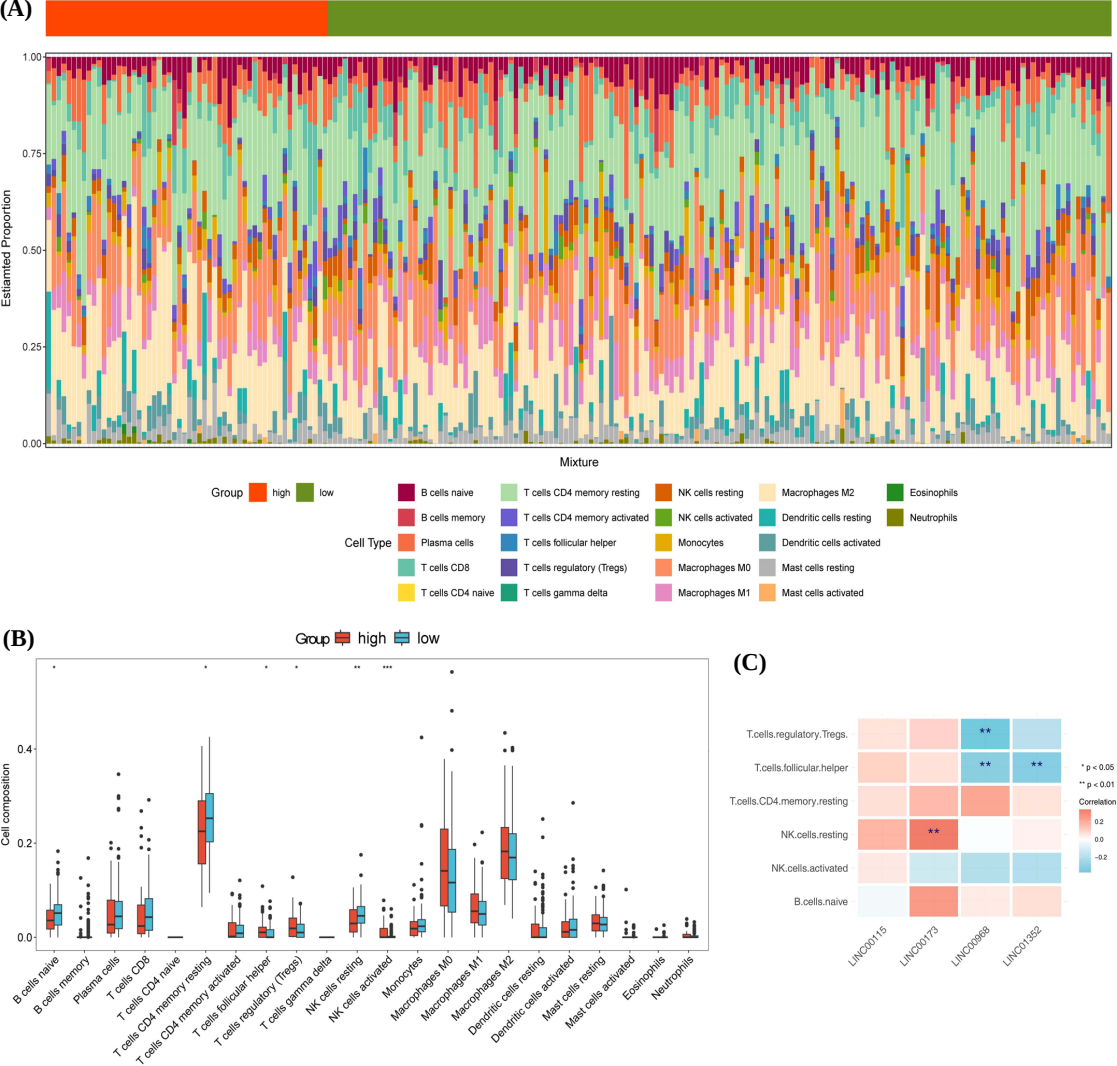
Immune cell analysis in the risk cohorts. (**A**) Stacked diagram depicting immune cell distributions in high- and low-risk groups based on CIBERSORT. (**B**) Box plot comparing immune cell levels between high- and low-risk groups. (**C**) Heatmap illustrating the relationship between immune cells and lncRNAs. *, *p* < 0.05; **, *p* < 0.01; ***, *p* < 0.001; ****, *p* < 0.0001.

**Fig. (7):**
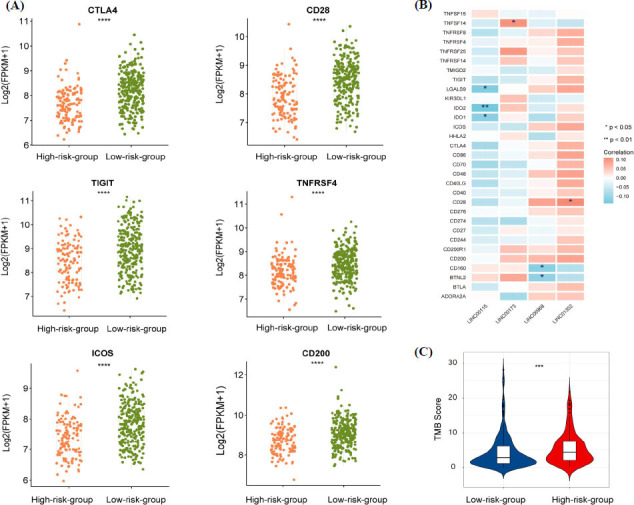
Prediction of immunotherapy response in risk cohorts. (**A**) Scatterplot illustrating the expression levels of immune checkpoint inhibitor (ICI) genes between high- and low-risk groups. (**B**) Correlation analysis of lncRNAs with ICI genes. (**C**) Differences in TMB scores between high- and low-risk groups. *, *p* < 0.05; **, *p* < 0.01; ***, *p* < 0.001; ****, *p* < 0.0001.

**Fig. (8):**
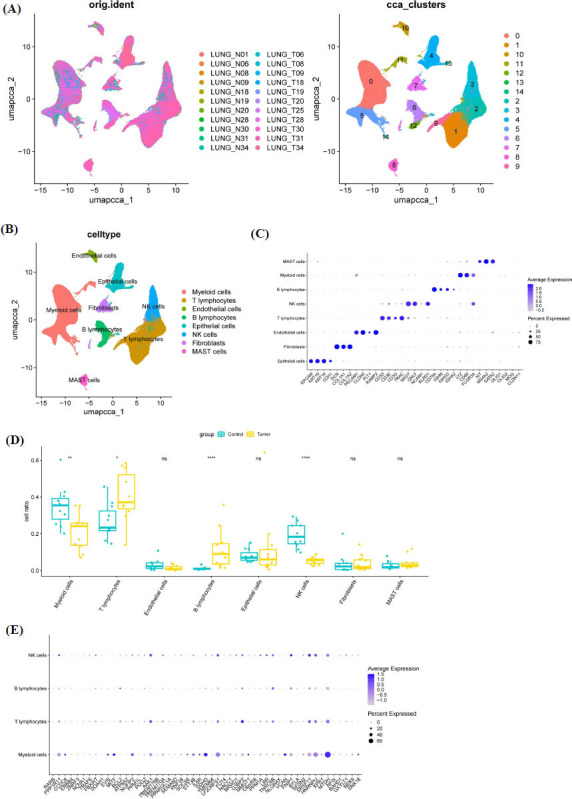
The identification of key cells. (**A-B**) Both the x-axis (UMAP_1) and y-axis (UMAP_2) represent abstract embedded dimensions derived from dimensionality reduction. Cells within the same cluster appear closer. (**A**) UMAP plots showing (left) tumor and normal samples, and (right) cell clusters. (**B**) Cell type identification with color coding for different cell types. (**C**) Expression of marker genes across various cell types. (**D**) Comparison of cell ratios between tumor and normal samples. (**E**) Key mRNA expression in differentiated cells. *, *p* < 0.05; **, *p* < 0.01; ***, *p* < 0.001; ****, *p* < 0.0001.

**Fig. (9):**
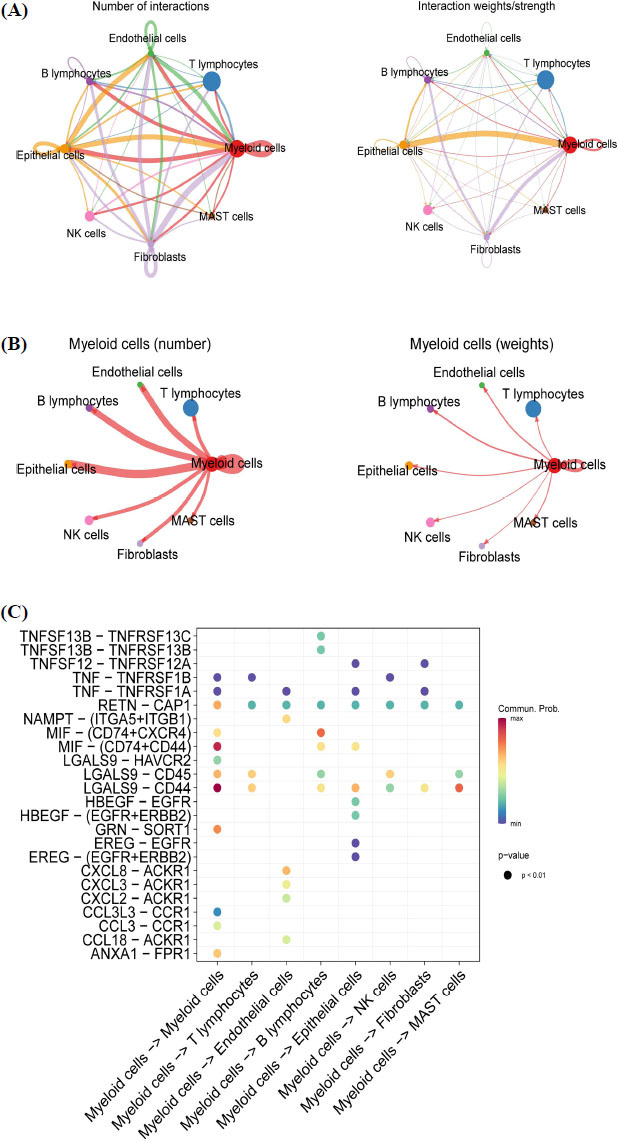
Analysis of cellular communication. (**A-B**) Left: the size of each colored circle in the periphery corresponds to the cell population count; larger circles indicate greater cell numbers. Cells emitting arrows express ligands, while cells receiving arrowheads express receptors. The thickness of connecting lines scales with the number of ligand-receptor pairs identified between cell clusters. Right: the probability score of cell-cell interactions (interaction strength is the sum of probability values; (**A**) (left) number and (right) strength of communication networks between different cell types; (**B**) interaction between myeloid cells and seven other cell types, with left representing number and right representing weight. (**C**) Bubble plot of ligand-receptor interactions between key cells and other cell types.

**Fig. (10):**
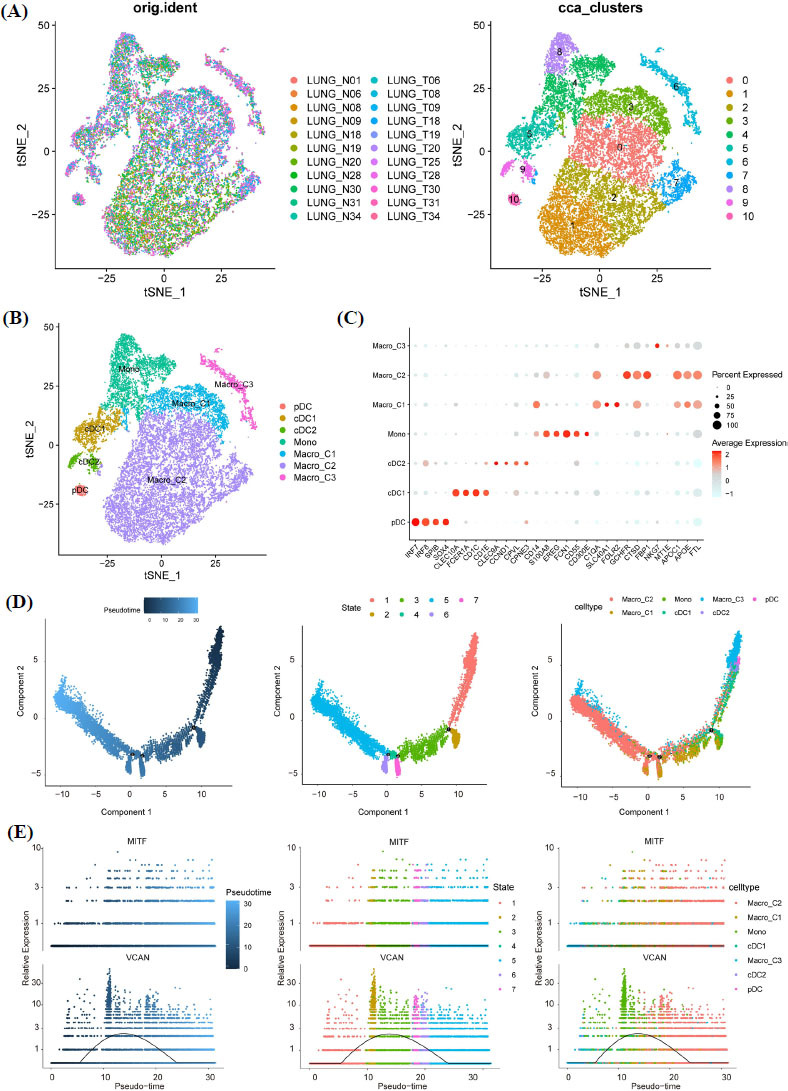
Comprehensive evaluation of the functional characteristics of various subcellular myeloid cell types. (**A-B**) The x-axis (t-SNE_1) and y-axis (t-SNE_2) represent the dimensions of t-distributed stochastic neighbor embedding, where cells within the same cluster are positioned closer to each other. (**A**) t-SNE plot depicting (left) myeloid cells in tumor and normal samples and (right) cell clusters. (**B**) t-SNE plot showing subcellular types associated with myeloid cells. (**C**) Expression of marker genes in subcellular myeloid cell types. (**D**) Developmental differentiation trajectories of key cells, with left representing pseudotime, and each dot represents a single cell. Numbered black circles indicate nodes corresponding to distinct cellular states in the trajectory analysis. Color gradient (dark-to-light blue) reflects pseudotime ordering, with dark blue representing early states and light blue denoting late states, middle representing stages, and right representing cell types. (**E**) Changes in the expression of key mRNAs at various stages of cellular differentiation. The x-axis represents pseudotime, while the y-axis shows gene expression levels in individual cells. The black line depicts the expression trend of the gene across pseudotime.

**Fig. (11):**
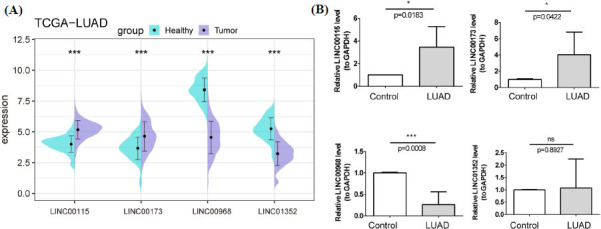
The expression of lncRNA in normal and tumor samples. (**A**) Variations in lncRNA expression profiles within the TCGA database. (**B**) RT-qPCR validation using normal and tumor samples. *, *p* < 0.05; **, *p* < 0.01; ***, *p* < 0.001; ****, *p* < 0.0001.

**Table 1:** Eight survival-associated genes identified by multivariate Cox analysis.

**Gene ID**	***Z***	***HR***	***HR*.95L**	***HR*.95H**	***p* value**
DDX11_AS1	2.82279269	1.33247555	1.09170163	1.62635197	0.00476073
LINC01352	-2.68729610	0.78886050	0.66355650	0.93782653	0.00720331
LHFPL3_AS2	-2.24181075	0.92053497	0.85625188	0.98964411	0.02497361
LINC00115	-2.23919975	0.79318336	0.64758331	0.97151956	0.02514292
LINC00968	-2.15436425	0.86522649	0.75846010	0.98702209	0.03121162
LINC00173	-2.06050487	0.85185362	0.73135416	0.99220682	0.03935030
EP300_AS1	-2.00962055	0.80720922	0.65504411	0.99472190	0.04447136
ADAMTS9_AS2	-1.99446829	0.87750673	0.77176074	0.99774195	0.04610090

**Table 2:** Four prognostic lncRNAs identified through multivariate Cox analysis and stepwise pairwise regression analysis.

**Gene ID**	***coef***	***HR***	***HR*.95L**	***HR*.95H**	***p* value**
LINC00115	-0.28850592	0.74938237	0.60511923	0.92803849	0.00818071
LINC00173	-0.15050618	0.86027241	0.73547197	1.00624994	0.05982885
LINC00968	-0.16553532	0.84743993	0.71741221	1.00103459	0.05143930
LINC01352	-0.17463863	0.83976043	0.68911765	1.02333408	0.08339882

## Data Availability

The LUAD data analyzed in this study are publicly available in the TCGA-LUAD dataset *via* the GDC Data Portal: https://portal.gdc.cancer.gov/. The gene expression data from GSE31210 and GSE131907 are available in the GEO database at https://www.ncbi.nlm.nih.gov/geo/ (accession numbers: GSE31210 and GSE131907). All custom R scripts used for data processing and analysis have been deposited in a publicly accessible GitHub repository: https://github.com/pengwuke/YQCQ.git.

## LIST OF ABBREVIATIONS

DE-lncRNAs: Differential Expression lncRNAs
GSEA: Gene Set Enrichment Analysis
lncRNAs: Long non-coding RNAs
LUAD: Lung Adenocarcinoma
NSCLC: Non-small Cell Lung Cancer
ROC: Receiver Operating Characteristic
SRDLs: Sialylation-related Difference lncRNAs
