# Supplementary material for: Unveiling the Role of Sialylation-Related LncRNAs in Lung Adenocarcinoma: A New Prognostic Model
Source: Curr Genomics. 2026 Apr 15;26(6):653–72. doi: 10.2174/0113892029382696251125102036 (PMC13154261; doi:10.2174/0113892029382696251125102036)
Supplement: Supplementary file 1 [file CG-26-6-653_SD1.pdf]

Supplementary Material

Unveiling the Role of Sialylation-Related LncRNAs in Lung Adenocarcinoma: A New Prognostic Model

Guixue Yang[1,#], Juncheng Yu[1,#], Xufeng Deng[1], Jigang Dai[1,*] and Quanxing Liu[1,2,*]

[1]Department of Thoracic Surgery, Xinqiao Hospital, Army Medical University (Third Military Medical University), Chongqing 400037, China; [2]Hematopoietic Acute Radiation Syndrome Medical and Pharmaceutical Basic Research Innovation Center, Ministry of Education of the People's Republic of China, Chongqing 400037, China

Table S1. The 38 common immune checkpoints obtained from previous studies.

ICI
CTLA4
CD160
ICOS
LGALS9
TNFRSF25
CD70
CD200
CD48
BTLA
IDO2
CD86
TNFSF14
TMIGD2
CD244
CD200R1
CD28
CD27
TNFRSF4
PDCDI
IDO1
CD40
TNERSF18
HHLA2
CD40LG
TNFSF15
TNFRSF8
BTNL2
LAG3
CD276
LAIRI
TNFSF4
KIR3DL1
TNFSF9
TNFRSF14
TIGIT
ADORA2A
CD274
NRPI

Table S2. The primer sequences of qPCR.

Primer	Sequence
LINC00115 F	AGCGGTGACTGTTGGGTG
LINC00115 R	CATCCACAGCGAGGCAAT
LINC00173 F	CCTCCCCAGCACCTAGTCTT
LINC00173 R	AGGATAAATGACCCCAGGCA
LINC00968 F	GTCCACCCACTGGTCCATTT
LINC00968 R	GTGCTGAGCTGTCTGGAAGT
LINC01352 F	ATGGGTGAACCCCAACACAC
LINC01352 R	GCTGATGAGGAAGCTTTGCC
internal reference H-GAPDH F	CGAAGGTGGAGTCAACGGATTT
internal reference H-GAPDH R	ATGGGTGGAATCATATTGGAAC

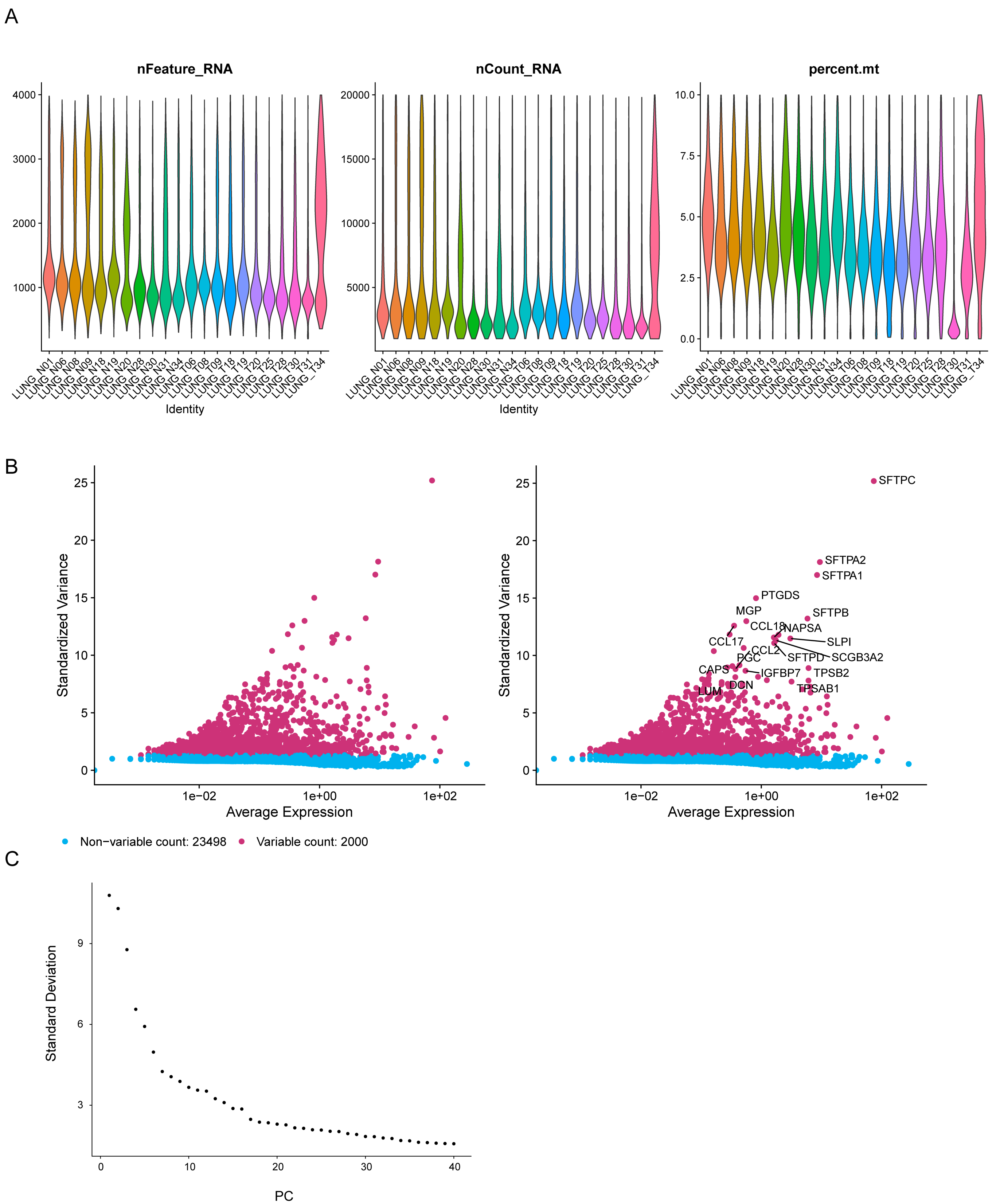

Fig. (S1). Single-cell data preprocessing. (**A**) Assessment of quality control; (**B**) data integration and standardisation; (**C**) linear optimal dimension values for cell clustering.

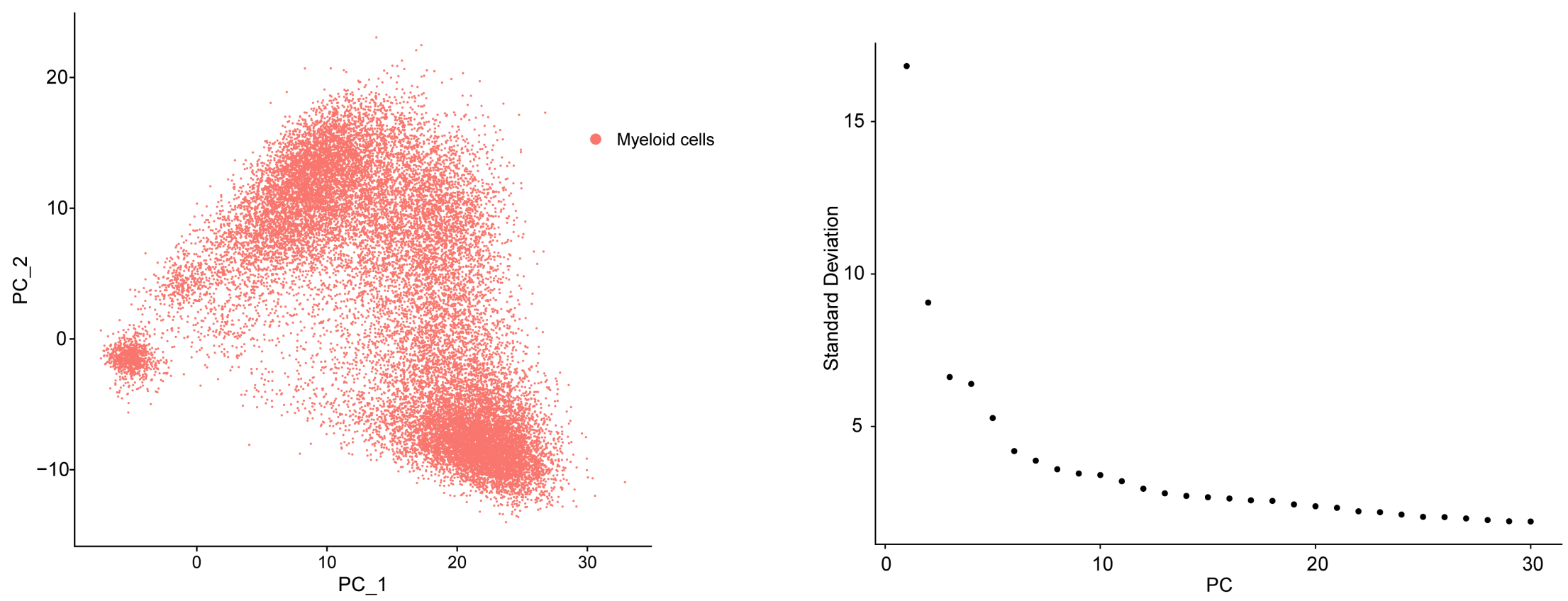

Fig. (S2). Annotation of subcellular myeloid cell types. (Left) PCA plot of dimensionality reduction analysis and (right) linear optimal dimension values for cell clustering.
